# Slow identification of facial happiness in early adolescence predicts onset of depression during 8 years of follow-up

**DOI:** 10.1007/s00787-016-0846-1

**Published:** 2016-04-22

**Authors:** Charlotte Vrijen, Catharina A. Hartman, Albertine J. Oldehinkel

**Affiliations:** Interdisciplinary Center Psychopathology and Emotion regulation, Department of Psychiatry, University of Groningen, University Medical Center Groningen, CC72, PO Box 30001, 9700 RB Groningen, The Netherlands

**Keywords:** Adolescents, Depression, Anhedonia, Facial emotion recognition, Risk factors, Reward system

## Abstract

Adolescent onset depression places a high burden on those who suffer from it, and is difficult to treat. An improved understanding of mechanisms underlying susceptibility to adolescent depression may be useful in early detection and as target in treatment. Facial emotion identification bias has been suggested as trait marker for depression, but results have been inconclusive. To explore whether facial emotion identification biases may be trait markers for depression, we tested whether the speed with which young adolescents identified happy, sad, angry and fearful facial emotions predicted the onset of depression during an eight-year follow-up period. We hypothesized that facial emotion identification speed predicts depression in a symptom-congruent way and differentially predicts symptoms of anhedonia and sadness. Data were collected as part of the TRacking Adolescents’ Individual Lives Survey (TRAILS), and involved 1840 adolescents who participated in a facial emotion identification test at age 11 and were subjected to the World Health Organization Composite International Diagnostic Interview (CIDI) at age 19. In a multi-emotion model, slow identification of happy facial emotions tentatively predicted onset of depressive disorder within the follow-up period. Slow identification of happy emotions and fast identification of sad emotions predicted symptoms of anhedonia, but not symptoms of sadness. Our results suggest that the relative speed of identification of happiness in relation to the identification of sadness is a better predictor of depression than the identification of either facial emotion alone. A possible mechanism underlying the predictive role of facial emotion identification may be a less reactive reward system.

**Electronic supplementary material:**

The online version of this article (doi:10.1007/s00787-016-0846-1) contains supplementary material, which is available to authorized users.

## Introduction

Adolescent unipolar depression is a common and major mental health problem. In mid to late adolescence, the estimated 1 year prevalence is 4–8 % worldwide [1–3], and at the end of adolescence, the estimated cumulative incidence is 16–28 % in community samples [2, 3]. For adolescents aged 10–19, depression is the first leading cause of disability as measured in disability-adjusted life years (DALYS) [4], and is strongly linked to suicide risk [5]. According to the DSM [6], the two core symptoms of depression are anhedonia (loss of pleasure) and sadness; experiencing either one of these symptoms is a necessary condition for receiving the diagnosis major depressive disorder (MDD). The burden of adolescent depression is not limited to adolescence: depression in adolescence is a strong predictor of adult depression. Experiences of subclinical symptoms of anhedonia and sadness during adolescence also predict adult depression, with stronger evidence for anhedonia [7, 8] than for sadness [8]. Depression in adolescence is often not recognized and not adequately treated [9]. Therefore, it is important to uncover mechanisms underlying susceptibility to adolescent depression and its core symptoms, to better understand the disorder. Ultimately, improved understanding may be useful in early detection and as target in treatment. Facial emotion processing^1^ is critical for normal emotional development and engaging in social relationships, and has been implicated as potential susceptibility factor for depression. The aim of the current study is to explore facial emotion processing bias in early adolescence as a potential trait marker for depression and symptoms of anhedonia and sadness in middle and late adolescence.

The body of knowledge concerning the connection between facial emotion processing and depressive symptoms is rapidly expanding, but results are highly heterogeneous. Research so far has largely been guided by cognitive theories claiming that negative cognitions initiate, maintain and strengthen depressive schemas [10], and by network models of emotion [11, 12] claiming that mood-congruent stimuli are processed more easily and correctly than mood-incongruent stimuli. According to these theories, depressed individuals are expected to suffer from negative biases in virtually all types of information processing, including perception, attention and memory. From the perspective of contemporary cognitive neuropsychological models of depression, which attempt to reconcile the cognitive theory with neurobiological findings, emotion processing bias is essential in understanding the mechanisms of depression. These biases have been claimed to be present before mood starts to deteriorate, and to be the main operating mechanism of depression treatment. It has been postulated that treatment enhances mood only indirectly through changing emotion processing biases, which in their turn instigate further changes that ultimately lead to improvement of mood [13, 14]. This suggests a direct relevance of measuring and monitoring emotion processing biases. Claims have also been made about the specificity of the emotion biases that are related to depressive symptoms. The content-specificity hypothesis states that depressed persons demonstrate stronger biases for themes that are consistent with depressed disorder, e.g., sadness and loss, than for anxiety-related stimuli such as threat and anger [15].

So far, empirical studies about facial emotion processing have been unable to provide conclusive empirical support for or against the above theoretical frameworks. Many studies have indicated relationships between biases in facial emotion information processing and depressive symptoms, but their findings have been far from consistent [16–20]. This can at least partly be explained by small sample sizes and varying measurements, instruments and experimental procedures [21, 22]. Another plausible cause is the heterogeneity of depressed patient groups [23]. When MDD patients are studied, usually no specific categories of depression are differentiated, and most studies do not address comorbidity issues. Despite evidence that the two core symptoms of depression according to the DSM [6], i.e., anhedonia and sadness, are associated with distinct psychophysiological systems reflecting approach and avoidance tendencies [24, 25], so far these core symptoms have not been used to differentiate between different types of depressive patients with respect to facial emotion processing. Because earlier studies did not distinguish between anhedonia and sadness and often not between depressed patients with and without anxiety disorders either, they were not equipped to test symptom- or content-specificity.

Another unresolved issue is that because of a lack of longitudinal studies, it is not clear whether facial emotion processing biases precede depressive symptoms or the other way around, and whether these relationships are trait- or state-dependent. Biases that are only present during depressive episodes suggest state dependency; biases that are also found preceding a depressive episode or after remission rather reflect traits. Only a limited number of studies have been published on facial emotion processing bias as a potential trait marker for depression, by investigating first-degree relatives or recovered depressed patients. Studies in first-degree relatives of depressed patients found that a familiar risk for depression increased 7–13-year-old boys’ ability to identify sad emotions [26], and that negative mood induction generated an attentional bias towards sad faces in 9–14-year-old girls with a familiar risk for depression, while girls without familiar risk showed a bias towards happy faces [27]. Studies involving recovered depressed patients showed that biases towards negative facial emotions [28], away from positive emotions [29], or both [30] persisted after remission. Furthermore, a study in young adults revealed that past depression (trait) was associated with greater salience of sad target faces; whereas current dysphoria (state) was related to a failure to inhibit responses to sad distractor faces [31]. Hence, empirical evidence regarding trait-dependent relationships between facial emotion identification and depression remains inconclusive and further research is needed.

Both facial emotion identification skills and onset of depression have been consistently reported to differ between males and females: the available evidence suggests a small female advantage in facial emotion identification throughout life [32], and a higher prevalence of depression in females than males starting in adolescence [33]. Gender differences have also been suggested in the relationships of facial emotion processing with depression [19, 26], but most were not suitable to test these gender differences statistically because of small samples. The one study that did test gender differences [19], found that facial emotion identification was associated with depression only in females. Altogether, this advocates considering gender when studying associations between facial emotion identification and depression.

We addressed several of the issues described above by performing a longitudinal study with a large sample size, differentiating between the two core depression symptoms anhedonia and sadness, and taking into account comorbid anxiety diagnoses. Focusing on facial emotion processing as a potential trait marker for depression, we investigated whether differences in emotion identification speed predicted later onset of depressive disorder and whether they differentially predicted symptoms of anhedonia and sadness. Major depressive disorder, minor depressive disorder and dysthymia were taken together in the overarching construct ‘depressive disorder’, since recent findings suggest that minor depressive disorder and dysthymia represent the same pathology as major depressive disorder, and differences among the disorders concern severity rather than qualitative characteristics [34–36].

We tested the following hypotheses:

(1) Speed in facial emotion identification at age 11 predicts the onset of later depressive disorder. (1a) Based on theories of mood congruence we expected that depression onset is predicted by slower identification of happy emotions and faster identification of sad emotions. This will be referred to as ‘symptom congruence’.^2^ We also expected to find (1b) content-specificity, i.e., stronger associations with happiness and sadness than with anger and fear. Considering the core symptoms anhedonia and sadness separately, we hypothesized that: (2a) slower identification of happy facial emotions is more predictive of anhedonia than of sadness; (2b) faster identification of sad emotions is more predictive of sadness than of anhedonia; (2c) fearful and angry face identification predict neither anhedonia nor sadness. Because of the inconsistent results of previous studies and tentative indications of the relevance of considering multiple emotions simultaneously [19]), we considered single-emotion models as well as multi-emotion models.

All associations were tested both regardless of comorbid anxiety and after exclusion of individuals with (lifetime) social phobia (SP) or generalized anxiety disorder (GAD), to explore to what extent the associations, if any, were depression-specific. SP and GAD were selected because of their social orientation, high comorbidity with depressive symptoms [37] and earlier evidence of associations between SP or GAD and facial emotion processing biases [18, 38]. Because of the plausibility of gender-specific relationships between facial emotion identification measures and onset of depression, we also tested gender interactions.

## Method

### Sample and procedure

This study is based on data collected as part of the TRacking Adolescents’ Individual Lives Survey (TRAILS), an ongoing cohort study investigating mental health and social development from early adolescence into adulthood. The study consists of two prospective cohort studies, a population-based cohort (*N* = 2230) and a clinical cohort (*N* = 543). TRAILS was approved by the Dutch Central Committee on Research Involving Human Subjects (CCMO), participants were treated in accordance with the Declaration of Helsinki, and written consent was acquired from all adolescents and their parents.

The data collection in both cohorts involved largely the same measures and participants were assessed at largely the same ages, every two or three years [39]. The specific questionnaires and tasks used were described in a previous report [39]. For the present study, we used data from the first (T1) and fourth (T4) waves of both cohorts. The participants of the population cohort were recruited from primary schools (response rate 90 %) in five municipalities in the northern region of the Netherlands. Of all eligible children, 2230 (76 %) agreed to participate. For more details on the selection procedure see De Winter and colleagues [40]. At T1, which ran from March 2001 until July 2002, the mean age of the population cohort was 11.1 years (SD 0.6), and 51 % were females. At T4 (from October 2008 until September 2010), 1881 adolescent participated again (retention rate 84 %), the mean age was 19.1 years (SD 0.6), and 52 % were females. Participants of the clinical cohort had been in contact with a specialized mental health service in the north of the Netherlands before the age of ten. Of all eligible participants asked, 543 (43 %) agreed to participate in the study. As was expected, non-response in this particular group was larger than it was in the population cohort. However, no significant differences were found between responders and non-responders in age, gender, parental education, age of referral to mental health services, teacher reports on mental health and on school achievement (except for lower mathematics performance in non-responders) [41]. At T1 (from September 2004 until December 2005), the mean age of the clinical cohort was 11.1 years (SD 0.5) and 34 % were females; at T4 (from September 2012 until April 2014), 422 adolescents participated again (retention rate 78 %), the mean age was 19.1 years (SD 0.7), and 34 % were females. The larger proportion of boys compared to girls in the clinical cohort is due to the fact that children with pervasive developmental disorder, attention deficit/hyperactivity and externalizing problems are referred to mental health services more often than those with internalizing problems [42, 43], and these problems are more common in boys than in girls [43–45].

From both cohorts, we selected all participants who: (1) completed the facial emotion identification task at T1; (2) had been subjected to the World Health Organization Composite International Diagnostic Interview (CIDI) at T4; and (3) had not had a depressive disorder, i.e., major depressive disorder, minor depressive disorder or dysthymia, as measured by the CIDI retrospectively at T4, prior to taking the facial emotion identification task. This yielded a sample of 1840 participants (81 % of the remaining population cohort at T4, 76 % of the remaining clinical cohort at T4).

Since the TRAILS study covered numerous research questions, no a priori power analysis was performed regarding our specific research question. For the present study, a post hoc power analysis for logistic regression [46] with an alpha set to 0.05, 1840 included participants, a proportion of lifetime depressed participants of 0.20, and a predefined effect of 20 % increased risk of depressive disorder per SD increase in the predictor variable, yielded an estimated power of 0.88. To detect an effect of 10 % increased risk the power decreased to 0.37. For outcomes with a proportion of about 0.35, like lifetime symptoms of anhedonia and sadness, the estimated power for effects of 20 and 10 % increased risk was, respectively, 0.96 and 0.49.

### Measures

#### Facial emotion identification

Facial emotion identification was measured by means of the ‘Identification of Facial Expressions’ (IFE) task at T1. This task was the last of seven tasks selected from the Amsterdam Neuropsychological Tasks program (ANT) [47], which in total took approximately 70 min to complete. Detailed information on the ANT testing procedures and the IFE task is provided in Online Resource 1.

Our hypotheses concerned the facial emotions happiness, sadness, anger and fear. Participants were included if they had completed the IFE task on at least one of these four emotions. For each of the four emotions we calculated the error proportion (EP) and the reaction time (RT). EPs were calculated as the mean proportion of misses and false alarms: ${\text{EP}} = \left( {\left( {{\text{misses}}/\left( {{\text{misses}} + {\text{hits}}} \right)} \right) + \left( {{\text{false alarms}}/\left( {{\text{false alarms}} + {\text{correct rejections}}} \right)} \right)} \right)/ 2.$ RTs were calculated by the mean RT across hits and correct rejections. Subsequently, EPs and RTs of more than four standard deviations above the mean [48] or EPs indicating performance at chance level, i.e., of 50 % or higher, were considered outliers and treated as missing. Because EP and RT potentially influence each other, outliers in one outcome parameter were also considered missing in the other. The percentage of missing EPs and RTs, including outliers, was less than 1.3 % for each facial emotion.

#### Depressive disorder and symptoms of anhedonia and sadness

At wave T4, the World Health Organization Composite International Diagnostic Interview (CIDI) version 3.0 [49] was used to assess onset of psychiatric disorders. The CIDI is a structured diagnostic interview which has been shown to have good reliability and validity in assessing current and lifetime DSM-IV disorders [50–52]. The interview started with a screening section for all participants, meant to determine which of the subsequent sections on specific disorders should be included in the interview. For each of these specific disorders age of onset was also registered.

In the present study, we were interested in the following outcome measures: (1) depressive disorder and (2) symptoms of anhedonia and sadness regardless of depressive disorder. The screening part of the CIDI depression section contained three questions: (1) ‘Have you ever in your life had a period lasting several days or longer when most of the day you felt sad, empty or depressed?’; (2) ‘Have you ever had a period lasting several days or longer when most of the day you were very discouraged about how things were going in your life?’; (3) ‘Have you ever had a period lasting several days or longer when you lost interest in most things you usually enjoy like work, hobbies, and personal relationships?’. All participants who endorsed at least one of these symptoms entered the whole depression section of the CIDI, which allowed classifying the participants according to DSM-IV criteria for major depressive disorder, minor depressive disorder, dysthymia, recurrent brief depression, and bipolar disorder. For the present study, depressive disorder was operationalized as the occurrence of at least one of the following disorders: major depressive episode, minor depressive disorder or dysthymia, and first onset of depressive disorder as the first onset of any of these three disorders. Since we were interested in predicting the incidence of depressive disorder after T1, we excluded the participants whose age of onset of depressive disorder was lower than or equal to their age at the time they took the facial emotion identification test at T1.

Within depressive disorders, correlations between anhedonia and sadness are high (in our sample, *r* = 0.78, *p* < 0.001), leaving little power to test anhedonia and sadness separately. Moreover, subclinical expressions of anhedonia and sadness were considered informative too. Therefore, we used CIDI screening items to determine the presence of symptoms of anhedonia and sadness regardless of the diagnostic status. Anhedonia was measured by the item ‘Have you ever had a period lasting several days or longer when you lost interest in most things you usually enjoy like work, hobbies, and personal relationships?’, and sadness by the item ‘Have you ever in your life had a period lasting several days or longer when most of the day you felt sad, empty or depressed?’. The correlation between these items was 0.37 (*p* < 0.001).

Since we were interested in predicting the incidence of symptoms of anhedonia and sadness after T1, we omitted participants who had already reported these symptoms in the Youth Self-Report (YSR) [53] at T1, when they were asked to report about the 6 months prior to T1. More specifically, we excluded participants with high scores (i.e., ‘clearly or often’) on T1 YSR item ‘I enjoy very little’, and participants with high scores (i.e., ‘clearly or often’) on T1 YSR item ‘I am sad, unhappy or depressed’ from the regression analyses of symptoms of anhedonia or sadness on facial emotion identification speed.

### Statistical analysis

Using SPSS version 22.0, we performed a series of logistic regression analyses to determine whether facial emotion identification speed (RTs) predicted onset of depressive disorder, anhedonia and sadness. Facial emotion identification can be assessed by RTs (speed) and by EPs (accuracy). We focused on speed rather than accuracy, because the task used (static facial emotion expressions presented at full intensity) is relatively easy for 11-year-old adolescents, and we therefore expected that identification speed would have more discriminative power than identification accuracy. To account for possible associations between speed and accuracy, we adjusted for accuracy.

Standardized RTs were used to be able to compare odds ratios (ORs) across different emotions. All analyses consisted of two steps: first, the effects of the RTs for happy, sad, angry and fearful emotions were tested separately, adjusted for the respective EPs, gender, and age at the time of the IFE task. Second, we started with a full model including the EPs and RTs for all facial emotions and ran a backward conditional logistic regression analysis (again adjusting for gender and age) to estimate the combined effect of emotion identification speed of multiple emotions. In the final models, we always adjusted for the EPs of all of the RTs in the model, to ensure that found effects could not have been driven by EPs rather than RTs.

In the first step of our analysis, i.e., testing the emotions separately, significance was set at 0.05 and in the second step, i.e., backward conditional logistic regression analyses, the entry criterion was set at 0.05 and the removal criterion at 0.10. The choice of a backward rather than a forward selection procedure was motivated by the idea that forward selection involves a higher risk of excluding predictors with a suppressor effect (i.e., predictors that are only significant if certain other predictors are included in the model as well), which we did not want to ignore beforehand because of the exploratory nature of this part of our study. The exploratory nature of this study was also the reason for choosing a backward conditional logistic regression removal criterion of 0.10 and not correcting for multiple tests in our initial analyses. The latter also implies that our results should not be interpreted in a formal discriminatory way, which is why we did not focus on single significant results but on more general patterns. The False discovery rate (FDR) method [54] was employed post hoc to give an indication which effects meet multiple test correction criteria. The maximum acceptable FDR was set to 0.05.

Since our analyses on the core symptoms anhedonia and sadness were primarily aimed at identifying symptom-specific facial emotion identification patterns, the effects of anhedonia and sadness were corrected for each other in these models. For the purpose of identifying potential gender differences, gender*RT interactions were tested for all separate emotion models. Practical limitations prohibited the inclusion of interactions with gender in the multi-emotion backward selection models.

Several additional specificity and sensitivity analyses were performed. To check if findings pertained specifically to depression all associations were tested both regardless of comorbid anxiety and after exclusion of all participants with T4 retrospective CIDI-based lifetime diagnoses of SP or GAD. Finally, we checked whether adjusting for baseline speed or cohort status (population cohort or clinical cohort) changed the main results.

## Results

### Number of participants excluded per exclusion criterion

From the total sample of 1921 participants who had been subjected to the IFE task at T1 as well as to the CIDI interview at T4, we excluded 81 participants with a CIDI depressive disorder first onset at the same time or prior to taking the IFE task. For the analyses concerning symptoms of anhedonia and sadness, we further excluded 45 participants with high scores (i.e., ‘clearly or often’) on T1 YSR item ‘I enjoy very little’, and 35 participants with high scores (i.e., ‘clearly or often’) on T1 YSR item ‘I am sad, unhappy or depressed’. The percentage of missing EPs and RTs, including outliers, was less than 1.3 % for each facial emotion: for happy we excluded 14 participants, for sad 10 participants, for angry 20 participants and for fearful 22 participants.

For the regression analyses of depression on facial emotion identification this yielded the following samples sizes: for RT Happy *N* = 1826, for RT Sad *N* = 1830, for RT Angry *N* = 1820, for RT Fearful *N* = 1818 and for the backward selection model *N* = 1785. And for the regression analyses of anhedonia and sadness on facial emotion identification the following: for RT Happy *N* = 1738, for RT Sad *N* = 1742, for RT Angry *N* = 1733, for RT Fearful *N* = 1733 and for the backward selection model *N* = 1701. For the additional specificity analyses we excluded 290 participants with CIDI-based lifetime diagnoses of SP or GAD.

### Descriptive statistics

Descriptive information of the variables used in this study is presented in Table 1. Between age 11 and age 19, 19 % of our sample developed a first depressive disorder, 35 % experienced symptoms of anhedonia and 42 % experienced symptoms of sadness. Female incidence rates were higher than male rates for depression and sadness, but not for anhedonia. Frequencies are in accordance with the findings from previous studies that in adolescence more females than males get depressed [33].

**Table 1:** Descriptives

Variables	Frequencies (%)/mean (SD)		
	Total sample (*N* = 1818–1840)	Males (*N* = 896–909)	Females (*N* = 922–931)
Depression^a^	344 (19 %)	111 (12 %)	233 (25 %)
Anhedonia^b^	618 (35 %)	306 (35 %)	312 (34 %)
Sadness^c^	765 (42 %)	318 (36 %)	447 (49 %)
RT Baseline^d^	334 (49)	332 (46)	336 (52)
RT Happy^d^	878 (206)	883 (213)	874 (199)
RT Sad^d^	1210 (287)	1229 (299)	1191 (274)
RT Angry^d^	1117 (259)	1125 (271)	1108 (245)
RT Fearful^d^	1112 (277)	1119 (282)	1105 (272)
EP Happy^e^	3.3 (3.3)	3.5 (3.4)	3.1 (3.3)
EP Sad^e^	12.6 (9.2)	13.4 (9.4)	11.9 (9.0)
EP Angry^e^	8.4 (6.1)	8.6 (5.9)	8.1 (6.3)
EP Fearful^e^	7.5 (6.6)	7.9 (6.8)	7.2 (6.4)

^a^CIDI-based DSM-IV diagnosis of major depressive disorder, minor depressive disorder or dysthymia, with age of onset between 11 and 19

^b^Symptoms of anhedonia for at least several consecutive days between age 11 and 19

^c^Symptoms of sadness for at least several consecutive days between age 11 and 19

^d^ *RT* mean reaction time for correct responses measured in milliseconds, assessed at age 11

^e^ *EP* mean error proportion, assessed at age 11

Table 1 shows differences in reaction times (RTs) of identifying the different facial emotions. Happy emotions were identified faster (shorter RTs) than the other facial emotions and participants had most difficulties with identifying sad facial expressions (longer RTs). Females identified facial emotions faster than males. Correlations between RTs (data not presented in table) varied from *r* = 0.61, *p* < 0.001 (RT Happy and RT Fear), to *r* = 0.74, *p* < 0.001 (RT Angry and RT Sad). See Online Resource 2 for descriptive statistics of facial emotion identification RTs and EPs by emotion by diagnostic group.

### Prediction of onset of depressive disorder by facial emotion identification RTs (Table 2, left side)

**Fig. 1:**
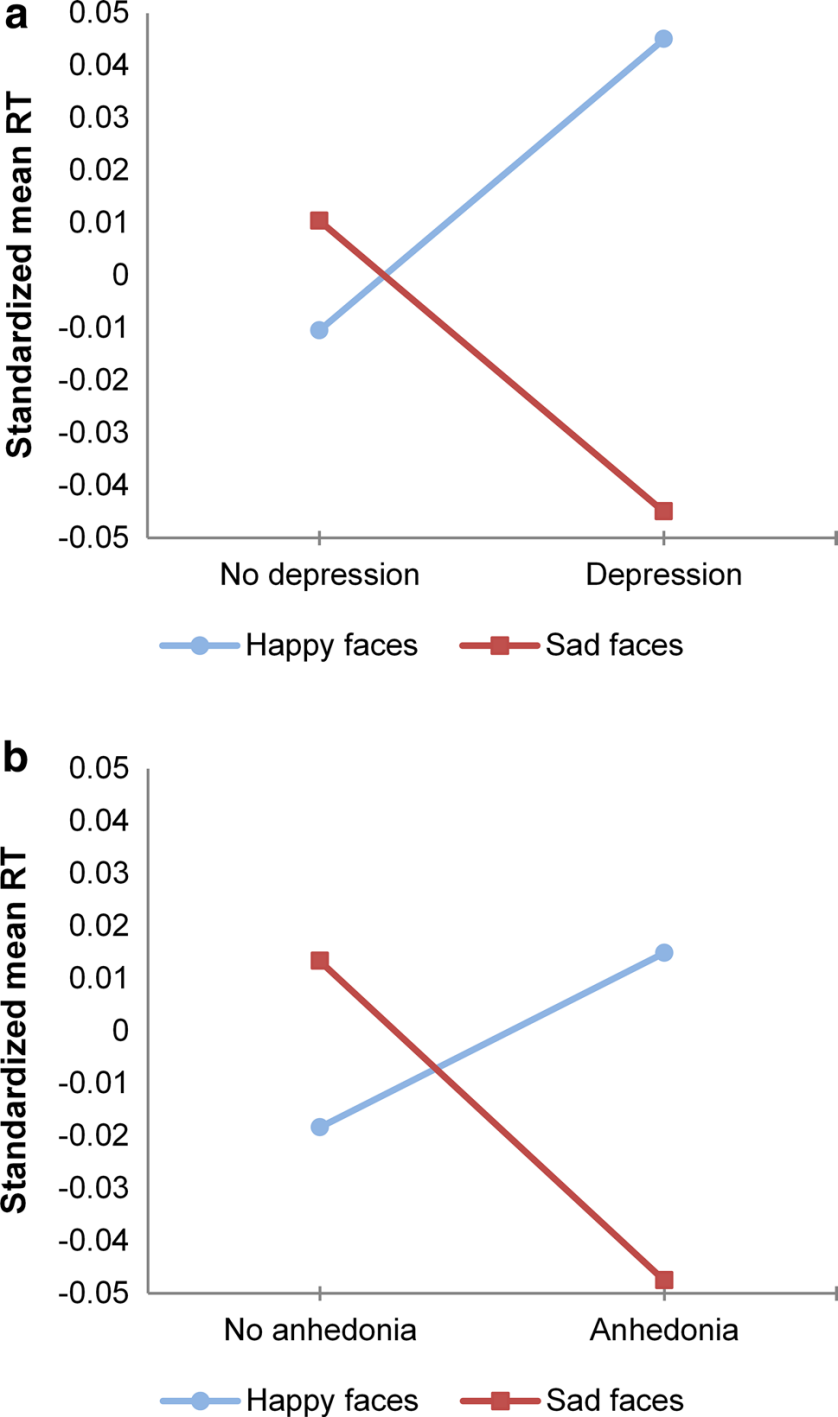
Standardized reaction times (RTs) for the identification of happy and sad facial emotions, for young adolescents with and without onset of depressive disorder during the 8-year follow-up period (**a**), and for those with and without symptoms of anhedonia during the follow-up period (**b**) (please note that standardized RTs are presented in this figure. With respect to the absolute values, for those who later develop depression or experience symptoms of anhedonia the group mean of RT Happy is still lower than the group mean of RT Sad.)

Facial emotion identification RTs at age 11 did not significantly predict onset of depressive disorder when testing each facial emotion separately. Backward conditional logistic regression analysis resulted in a multi-emotion model in which onset of depression was predicted by longer RTs (OR greater than 1) for happiness in combination with shorter RTs (OR smaller than 1) for sadness, of which only RT Happy reached statistical significance at *α* = 0.05. These results are graphically presented in Fig. 1a. Additional analyses revealed that the presence of RT Happy in the multi-emotion model relied specifically on the inclusion of RT Sad and the other way around; neither RT Happy nor RT Sad could be replaced by RT Fearful or RT Angry. Excluding either RT Happy or RT Sad from the model resulted in no longer finding any effect with backward model selection.

**Table 2:** Results logistic regression analyses of DSM-IV depression and symptoms of anhedonia and sadness for at least several days between age 11 and age 19 on facial emotion identification reaction times at age 11

	Depression (*N* = 1785–1830)^a^		Anhedonia^b^ with sadness as covariate (*N* = 1701–1742)		Sadness^c^ with anhedonia as covariate (*N* = 1701–1742)	
	OR	*P*	OR	*P*	OR	*P*
**Emotions tested separately**						
RT Happy	1.07	0.25	1.05	0.39	0.97	0.60
RT Sad	0.97	0.67	0.93	0.16	1.04	0.44
RT Angry	1.02	0.77	0.99	0.86	0.98	0.64
RT Fearful	0.96	0.54	1.01	0.87	1.04	0.51
**Models backward selection**						
RT Happy	1.19	0.04	1.22	0.009		
RT Sad	0.86	0.09	0.80	0.005		
RT Angry						
RT Fearful						
**Post hoc analyses**						
RT Sad – RT Happy (HS)	0.89	0.08	0.85	0.004	Not tested	Not tested

All effects were adjusted for error proportions, gender and age at the time of the facial emotion identification task

*OR* odds ratio, *RT* mean reaction time for correct responses; all RTs in this table are standardized (*Z*-values) with one exception: HS was calculated on unstandardized RT Sad and RT Happy and was standardized afterwards

^a^CIDI-based DSM-IV diagnosis of major depressive disorder, minor depressive disorder or dysthymia

^b^Symptoms of anhedonia for at least several consecutive days

^c^Symptoms of sadness for at least several consecutive days

### Prediction of symptoms of anhedonia and sadness by facial emotion identification RTs (Table 2, right side)

Facial emotion identification RTs did not predict anhedonia when each facial emotion was tested separately. Backward conditional logistic regression analysis resulted in a multi-emotion model in which anhedonia was predicted by longer RTs for happiness and shorter RTs for sadness (see also Fig. 1b), of which both RT Happy and RT Sad reached statistical significance. Again, additional analyses revealed that the presence of RT Happy in the multi-emotion model relied specifically on the inclusion of RT Sad and the other way around. Excluding either of them from the model resulted in no longer finding any effect with backward model selection.

No significant associations were found between facial emotion identification RTs and later symptoms of sadness, neither when emotion RTs were tested separately, nor when they were tested in a multi-emotion model.

In Table 2 symptoms of anhedonia were corrected for symptoms of sadness and the other way around. Additional analyses without correcting for anhedonia and sadness showed no important differences in results (for exact p-values and ORs see Online Resource 3).

### Multiple test correction

After FDR correction for multiple testing, the effect of RT Happy on depressive disorder was no longer significant (p_FDR-corrected_ = 0.28). Effects of RT happy (p_FDR-corrected_ = 0.03) and RT Sad (p_FDR-corrected_ = 0.03) on symptoms of anhedonia remained significant after FDR correction.

### Post hoc analyses (Table 2, bottom)

Post hoc analyses were performed in an attempt to explain the differences in results between emotions tested in separate models and in one multi-emotion model. Combined, the results of the single- and multi-emotion models (see Table 2; Fig. 1a, b) suggest that the intra-individual contrast between RT Happy and RT Sad is more relevant to predict onset of depressive disorder and symptoms of anhedonia than each RT individually.

To test the plausibility of this explanation, we constructed the variable: Happy/Sad specialization (HS) = RT Sad-RT Happy. A value of 0 indicated no Happy/Sad specialization, i.e., participants responded equally fast to happy and sad emotions. Positive values indicated a specialization in identifying happiness (in the sense of being faster) and negative values a specialization in identifying sad faces (again, in the sense of being faster). Subsequently, we checked whether the standardized new variable predicted depressive disorder and symptoms of anhedonia. As in previous analyses, we adjusted for relevant EPs, gender and age at the time of the IFE task. Table 2 (bottom) shows the results of the post hoc analyses. HS was found to predict symptoms of anhedonia significantly. The ORs of HS were below 1 which means that more specialization towards identifying happy faces was associated with decreased risk of developing anhedonia. The same pattern was found for depression, but HS did not reach statistical significance at *α* = 0.05.

### The effects of excluding SP and GAD participants

After excluding participants with lifetime diagnoses of SP or GAD, the effect of RT Happy on depression became slightly stronger and the effect of RT Sad weakened compared to the findings based on the complete sample. Regarding symptoms of anhedonia and sadness, excluding participants with SP or GAD diagnoses did not result in different patterns compared to the ones found in the whole sample, except for slightly stronger effects for anhedonia. For specific p-values and ORs of the analyses after excluding SP or GAD participants, see Online Resource 4.

### Gender differences

We did not find any significant RT Happy*gender, RT Sad*gender, RT Angry*gender or RT Fearful*gender effects for depression, anhedonia or sadness in the single-emotion models.

### Additional sensitivity analyses

Adjusting for baseline speed did not change results (data not presented). Adjusting for cohort status did not make a difference to the effect sizes, i.e., the ORs remained virtually the same, but the decrease in power was reflected in slightly higher *p*-values (see Online Resource 5).

## Discussion

The aim of this study was to examine whether facial emotion identification in early adolescence predicts onset of depressive disorder, whether it differentially predicts symptoms of anhedonia and sadness, and whether it does so in a symptom-congruent and content-specific way. Because of the exploratory nature of this study our main focus was on patterns rather than single results.

Our results provide tentative evidence in favor of the hypothesis that facial emotion identification in early adolescence predicts onset of depressive disorder and symptoms of anhedonia within eight-year follow-up. In support of the hypothesis of symptom congruence, both risk of depressive disorder and risk of anhedonia were associated with slower identification of happy emotions; risk of anhedonia was also associated with faster identification of sad emotions. However, we found no evidence for the hypothesis that symptoms of sadness are predicted by faster identification of sad emotions. In favor of the content-specificity hypothesis, identification of angry or fearful emotions predicted neither onset of depressive disorder, nor symptoms of anhedonia or sadness.

Our prospective findings suggest that facial emotion identification bias may be a symptom-congruent trait marker for depressive disorder and anhedonia. These associations were only found when considering multi-emotion models or Happy/Sad specialization. It seems primarily relevant how fast young adolescents identify happy facial emotions compared to how fast they identify sad emotions. Our results suggest that those who identify sad expressions faster than happy ones or are only relatively faster in identifying happiness seem more prone to developing depression or symptoms of anhedonia. Although largely similar patterns were found for depressive disorder and anhedonia, effects for depressive disorder did not meet multiple test correction criteria, and should therefore be interpreted with caution. Not finding results for symptoms of sadness implies that facial emotion identification is not a trait marker for sadness, but it could still be a symptom-congruent state marker for sadness. The content-specificity of the associations found implies that young adolescents’ identification of happy and sad facial emotions is more relevant for onset of depressive disorder and symptoms of anhedonia than identification of facial anger and fear.

Effects of facial emotion identification on depressive disorder seem to be mainly carried by symptoms of anhedonia and not by symptoms of sadness. Happy faces are naturally strongly rewarding stimuli. Since identifying happy faces less fast relative to sad ones seems to predict depression and anhedonia but not sadness, we propose that the mechanism underlying this vulnerability might be related to the functioning of the reward system. As mentioned in the introduction, symptoms of anhedonia and symptoms of sadness have been associated with partly different psychophysiological systems: the so-called approach (reward-related) and avoidance systems [24, 25]. Whereas the reward system is assumed to be an important underlying mechanism of the development of anhedonia, there is less evidence that it is also directly involved in the development of sadness. Being able to identify happy emotions much faster than sad ones may point at a more reactive reward system, whereas small differences between identifying these emotions, or even being faster in identifying sad emotions, suggest a more passive reward system. One of the mechanisms underlying depression and anhedonia may be an impaired tendency to modulate behavior as a function of prior rewarding experiences [55, 56]. Whereas individuals without vulnerability for depression have a tendency to approach rewarding stimuli (e.g., happy faces), those with a vulnerability for depression and anhedonia may not have developed this inclination. Blunted responsiveness to rewarding stimuli could contribute to loss of interest in the environment and in this way contribute to the onset of depression [57]. A bias towards sad faces and away from happy ones may also contribute to onset of depression via ineffective emotion regulation strategies. Attitudes of dwelling on negative feelings and avoiding positive cues that could help to overcome negative experiences have been found to predict depression [58]. This pathway is supported by recent findings that adolescents between ages 9 and 14 with ruminating response styles, who were unable to disengage from self-referential negative thoughts, were also characterized by an attentional bias away from happy faces [59].

We found no evidence for gender differences in the relation between facial emotion identification and later onset of depressive disorder or symptoms of anhedonia and sadness, but did find small differences in results depending on whether adolescents with lifetime social or generalized anxiety were in- or excluded. When anxious adolescents were excluded, the predictive value of facial emotion identification for depressive disorder and symptoms of anhedonia slightly strengthened. We are unable to explain the (small) differences in results and can only speculate that social or generalized anxiety disorder, which is correlated with depression and anhedonia, might be reversely associated with facial emotion identification. Including adolescents with social or generalized anxiety disorder may, therefore, have slightly concealed the association between facial emotion identification and risk of depression and anhedonia.

Overall our findings are consistent with the previous studies in which symptom-congruent and content-specific associations between facial emotion processing biases and depression were reported [18, 27, 60], but inconsistent with many other studies. This is not surprising since, as was already mentioned in the introduction, results of previous studies were quite diverse. Because many different measures for facial emotion identification and depression were used, it is difficult to compare our results to theirs. Furthermore, most previous studies looked at cross-sectional associations between facial emotion identification and depression; studies investigating facial emotion identification as a potential trait marker for depression are scarce. Our finding that the identification speed pattern across multiple emotions seems to be more relevant than identification speeds of individual emotions cannot be interpreted in relation to prior evidence, because—with the exception of only a few studies focusing on positive–negative bias [19, 61]—this field has remained largely unexplored so far. Hence, awaiting further support our conclusions are tentative.

Our study has several strengths. We are one of the very few who focused on predicting onset of depressive disorder and symptoms rather than on cross-sectional associations. This is the only way to shed light on the possibility of facial emotion identification differences as trait marker of depression or depressive symptoms, which could have implications for treatment and prevention. Our study has a large sample size and a follow-up period of 8 years, and depression and symptoms of anhedonia and sadness were assessed by means of standardized diagnostic interviews. The large sample size enabled us to test all associations regardless of comorbid anxiety and after exclusion of adolescents with lifetime social or generalized anxiety disorder, hereby ensuring internal validity as well as ecological validity. To our best knowledge, we were the first to examine symptoms of anhedonia and sadness separately in relation to facial emotion identification, and also the first to explore the possibility of Happy/Sad facial emotion identification specialization as predictor of depression, anhedonia and sadness.

However, our study is not without limitations. First of all, the inconsistent results reported in earlier studies, as well as the suppressor effects found in our own study, called for consideration of a large number of interrelated hypotheses and various (post hoc) options to find novel patterns. Suppressor effects, i.e., that single predictors were only significant if other predictors were included in the model as well, occur more frequently when predictors are highly correlated and are mainly due to large standard errors of the estimates, suggesting that suppression situations may be less replicable, and therefore, caution is needed [62]. We performed post hoc analyses in an attempt to explain differences in results between emotions tested separately and when tested together in one model, in the hope to demystify our suppressor findings. Although the post hoc analyses did provide clues to an explanation, the problem of capitalization on chance should be noted. Second, all effect sizes reported in this study are small. Although it can be argued that the effect sizes are reasonable considering the longitudinal nature of our study, and the fact that we corrected for early depressive diagnoses and symptoms, it is difficult to estimate the clinical relevance of our findings. Third, symptoms of anhedonia as well as symptoms of sadness were measured by single items from the CIDI screening list. The CIDI is not a survey but a structured diagnostic interview allowing for elaborate explanations and answers, but still the scores are based on one single question concerning anhedonia and one single question regarding sadness. Finally, the CIDI was assessed retrospectively at age 19. Although specific interview strategies were developed for the CIDI to reduce recall inaccuracies, e.g., decomposing questions and using specific life course events as point of reference [49], the possibility of recall biases cannot be excluded, especially for the earliest diagnoses and symptoms.

Further research is needed on many levels. First of all, our findings need to be confirmed by replication in other samples because of multiple testing and suppressor effects. Second, more research is needed into whether the results of this study can be generalized to older age groups and to clinical populations, since our sample consisted largely of young adults with mild psychiatric problems at the most. Related to this, participants indicated whether they had experienced anhedonia and sadness for ‘a period lasting several days or longer’, without additional information on the severity of the symptoms and on how many days they had experienced those symptoms. Although there is evidence that subclinical symptoms of anhedonia and sadness predict adult MDD [7, 8], more research is needed to determine to what extent mild symptoms of anhedonia and sadness belong to normative adolescent development and to what extent they reflect the characteristics that have predictive value for psychopathology. Third, in the facial emotion identification task used in this study participants were asked to judge full intensity facial emotions. It has been argued that, for the sake of ecological validity, more subtle emotions should also be taken into account, for example by using the so-called morphing tasks which show movies of neutral faces gradually changing into full intensity facial emotions. Fourth, our findings call for more focus on intra-individual multi-emotion patterns of facial emotion identification, a so far largely unexplored field of study. Furthermore, more research into possible underlying mechanisms is needed, e.g., reward responsiveness. As depression and anhedonia have already been linked to lower activity levels in reward-related brain areas during different stages of reward processing [55, 63, 64], a potentially viable direction for future research would be to determine whether emotion identification biases can also be linked to different responses in reward-related brain areas using fMRI methods. Finally, because of the small effect sizes found in our study, training facial emotion identification biases may not, in general, be expected to be efficient if used for treatment and prevention purposes. Training may, however, be effective if limited to adolescents with severe biases. Preliminary positive effects resulted from a randomized controlled trial of training the perception of happiness over sadness in ambiguous facial expressions [65], but more research is needed.

To conclude, from the perspective of contemporary cognitive neuropsychological models of depression according to which emotion processing biases are present before mood starts to deteriorate and mood is only enhanced via changing these emotion processing biases, measuring emotion processing bias and trying to modify these biases is of essence. Our findings point at a rather complex picture in which how fast young adolescents are able to identify happy facial emotions compared to how fast they identify sad emotions predicts onset of depression and symptoms of anhedonia (but not sadness) within a time frame of 8 years. A possible underlying mechanism could be a less reactive reward system.

## Electronic supplementary material

Below is the link to the electronic supplementary material.

Online Resource 1 (PDF 220 kb)

Online Resource 2 (PDF 92 kb)

Online Resource 3 (PDF 143 kb)

Online Resource 4 (PDF 94 kb)

Online Resource 5 (PDF 95 kb)

## Footnotes

1 In this paper, ‘facial emotion processing’ is used for reference to facial emotion processes that are not limited to facial emotion identification, but also entail attention, memory, etc. ‘Facial emotion identification’ will be reserved for the more specific concept of identification only.

2 ‘Mood congruence’ more commonly refers to a relationship between an identification bias and a concurrent mood state at one and the same time point. We stretched this use to a different time frame by hypothesizing the presence of a bias at one time point (age 11) to be congruent with depressive symptoms that are developed later (between age 11 and 19). To avoid misunderstandings, from now on we will no longer use ‘mood congruence’, but refer to this idea as ‘symptom congruence’.
