# Supplementary material for: Slow identification of facial happiness in early adolescence predicts onset of depression during 8 years of follow-up
Source: Eur Child Adolesc Psychiatry. 2016 Apr 22;25(11):1255–66. doi: 10.1007/s00787-016-0846-1 (PMC5083762; doi:10.1007/s00787-016-0846-1)
Supplement: Supplementary file 1 — Online Resource 1 (PDF 220 kb) [file 787_2016_846_MOESM1_ESM.pdf]

Article: Slow identification of facial happiness in early adolescence predicts onset of depression during eight years of follow-up
Journal: European Child & Adolescent Psychiatry
Authors: Charlotte Vrijen, Catharina A. Hartman, Albertine J. Oldehinkel
Affiliation: Interdisciplinary Center Psychopathology and Emotion regulation, Department of Psychiatry, University of Groningen, University Medical Center Groningen
Corresponding author: C. Vrijen@umcg.nl

Online Resource 1

Testing procedures of the Amsterdam Neuropsychological Tasks program (ANT) and the 'Identification of Facial Expressions' (IFE) task

The ANT was assessed in separate rooms at participants' schools or, if no separate rooms were available in participants' schools, at nearby community centers by trained undergraduate psychology students. To ensure that participants understood the instructions and kept in mind that both speed and accuracy of performance were of essence, practice trials were run prior to the test trials and screenshots of the tasks were explained verbally. The ANT started with a test to press a button as soon as a square was presented on the screen, in order to determine participants' baseline speed in simple cognitive decisions, and was followed by six other tasks (for a detailed description of the ANT in TRAILS, see Brunnekreef and colleagues [1]).

The IFE task, which was the last task of the ANT, was used to measure participants' capacity to identify different facial emotions [2]. Six different emotions were tested (happiness, sadness, anger, fear, disgust and surprise), each in a separate subtask by means of 40 trials, 20 trials in which the target emotion was presented and 20 nontarget trials in which a random selection of the other emotions was presented (hence, in total 240 trials). Each subtask consisted of a random sequence of target and non-target trials. Stimuli consisted of digitized high quality color photographs of four adult faces (two men and two women, frontal views) showing distinct expressions of one of the following six emotions: happiness, sadness, anger, fear, disgust and surprise (see Fig. 1 for examples of expressions of the emotions happiness and anger).

Fig. OR1

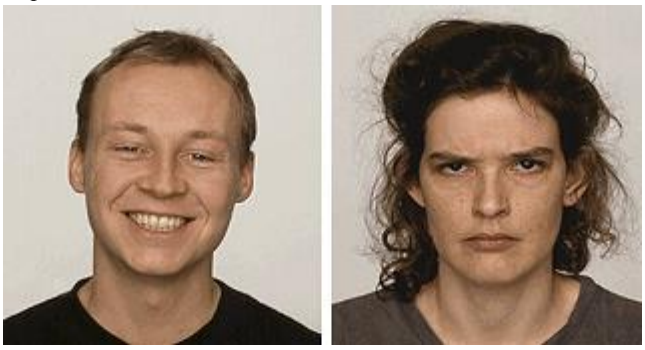

Examples of facial emotion expressions in the 'Identification of Facial Expressions' (IFE) Task. The left face expresses happiness and the right face expresses anger.

Each of the six emotion subtasks started with an instruction and practice session. Participants were shown an example of the target emotion and were instructed to focus on the target emotion and to respond whether the face showed the target emotion or not by clicking a mouse button. Participants had to press the 'yes' button (right button for right-handed participants and left button for left-handed participants) if the picture presented on a computer screen matched the target emotion and 'no' (left button for right-handed participants and right button for left-handed participants) if it did not. After the instruction, eight practice trials followed. Each subtask was preceded by showing a picture of the target emotion and each trial started with a presentation of a fixation cross for 500 ms, after which a stimulus face was presented on the screen until the participant pressed the 'yes' or the

‘no’ button. The inter-stimulus presentation time was 1000 ms and because of the variable presentation time of the stimuli, inter-trial intervals were also variable. Responses slower than 8000 ms were regarded invalid, as well as responses faster than 250 ms, which were interpreted as accidental responses. If participants did not press either of the answer buttons within 8000 ms or responded faster than 250 ms the trial was replaced by a new similar trial. The valid responses were coded as follows: ‘hit’ = correct ‘yes’ response; ‘correct rejection’ = correct ‘no’ response; ‘miss’ = no ‘yes’ response on target signal; ‘false alarm’ = ‘yes’ response on nontarget signal’. All participants were offered all emotion subtasks. Accidentally, participants were unwilling or unable to complete all subtasks, for instance because they did not understand the emotion that was tested.

References

1. Agnes Brunnekreef J, De Sonneville LMJ, Althaus M, et al. (2007) Information processing profiles of internalizing and externalizing behavior problems: evidence from a population-based sample of preadolescents. Journal of Child Psychology and Psychiatry 48:185–193. doi: 10.1111/j.1469-7610.2006.01695.x
2. De Sonneville LMJ (1999) Amsterdam Neuropsychological Tasks: A computer-aided assessment program. In: Cognitive ergonomics, clinical assessment and computer-assisted learning: computers in psychology. Swets & Zeitlinger, Lisse, pp 187–203
