# Supplementary material for: Slow identification of facial happiness in early adolescence predicts onset of depression during 8 years of follow-up
Source: Eur Child Adolesc Psychiatry. 2016 Apr 22;25(11):1255–66. doi: 10.1007/s00787-016-0846-1 (PMC5083762; doi:10.1007/s00787-016-0846-1)
Supplement: Supplementary file 2 — Online Resource 2 (PDF 92 kb) [file 787_2016_846_MOESM2_ESM.pdf]

Article: Slow identification of facial happiness in early adolescence predicts onset of depression during eight years of follow-up

Journal: European Child & Adolescent Psychiatry

Authors: Charlotte Vrijen, Catharina A. Hartman, Albertine J. Oldehinkel

Affiliation: Interdisciplinary Center Psychopathology and Emotion regulation, Department of Psychiatry, University of Groningen, University Medical Center Groningen

Corresponding author: C. Vrijen@umcg.nl

Online Resource 2

Descriptives for reaction times and error proportions at age 11 by diagnostic group between age 11 and 19

	Mean (*SD*)			
Variables	Depression[a] N = 342-344	Anhedonia[b] N = 614-617	Sadness[c] N = 758-764	Healthy[d] N = 797-800
RT Happy[e]	887 (216)	881 (203)	879 (208)	871 (206)
RT Sad[e]	1197 (284)	1196 (273)	1211 (302)	1207 (278)
RT Angry[e]	1117 (248)	1111 (257)	1115 (262)	1115 (258)
RT Fearful[e]	1100 (266)	1112 (278)	1116 (281)	1101 (273)
EP Happy[f]	3.0 (2.9)	3.1 (3.3)	3.2 (3.4)	3.4 (3.3)
EP Sad[f]	12.1 (8.8)	12.1 (8.8)	12.7 (9.1)	12.8 (9.4)
EP Angry[f]	8.1 (6.0)	8.1 (5.9)	8.2 (6.2)	8.5 (6.0)
EP Fearful[f]	7.2 (6.2)	7.2 (6.4)	7.4 (6.5)	7.6 (6.5)

[a] *CIDI-based DSM-IV diagnosis of major depressive disorder, minor depressive disorder or dysthymia, with age of onset between 11 and 19;*
[b] *Symptoms of anhedonia for at least several consecutive days between age 11 and 19;*
[c] *Symptoms of sadness for at least several consecutive days between age 11 and 19;*
[d] *No depressive disorder or symptoms of anhedonia or sadness;*
[e] *RT = mean reaction time for correct responses measured in milliseconds, assessed at age 11*
[f] *EP = mean error proportion, assessed at age 11*
