# Supplementary material for: Slow identification of facial happiness in early adolescence predicts onset of depression during 8 years of follow-up
Source: Eur Child Adolesc Psychiatry. 2016 Apr 22;25(11):1255–66. doi: 10.1007/s00787-016-0846-1 (PMC5083762; doi:10.1007/s00787-016-0846-1)
Supplement: Supplementary file 3 — Online Resource 3 (PDF 143 kb) [file 787_2016_846_MOESM3_ESM.pdf]

Article: Slow identification of facial happiness in early adolescence predicts onset of depression during eight years of follow-up

Journal: European Child & Adolescent Psychiatry

Authors: Charlotte Vrijen, Catharina A. Hartman, Albertine J. Oldehinkel

Affiliation: Interdisciplinary Center Psychopathology and Emotion regulation, Department of Psychiatry, University of Groningen, University Medical Center Groningen

Corresponding author: C. Vrijen@umcg.nl

Online Resource 3

*Results logistic regression analyses of DSM-IV depression and symptoms of anhedonia and sadness for at least several days between age 11 and age 19 on facial emotion recognition reaction times at age 11 – **without correcting for sadness and anhedonia***

		Anhedonia[a] ***without*** sadness as covariate N=1732-1773		Sadness[b] ***without*** anhedonia as covariate N=1748-1793	
		OR	P	OR	P
**Emotions tested separately**	RT Happy	1.04	.50	1.00	.95
	RT Sad	0.94	.24	1.02	.74
	RT Angry	0.97	.56	0.99	.80
	RT Fearful	1.02	.69	1.03	.53
**Models backward selection**	RT Happy	1.17	.03		
	RT Sad	0.84	.02		
	RT Angry				
	RT Fearful				
**Posthoc analyses**	RT Sad - RT Happy (HS)	0.88	.02	Not tested	Not tested

[a] *Symptoms of anhedonia for at least several consecutive days;*
[b] *Symptoms of sadness for at least several consecutive days;*
All effects were adjusted for error proportions, gender and age at the time of the facial emotion task;
OR = odds ratio; RT = mean reaction time for correct responses; all RTs in this table are standardized (Z-values) with one exception: HS was calculated on unstandardized RT Sad and RT Happy and was standardized afterwards
