# Supplementary material for: Slow identification of facial happiness in early adolescence predicts onset of depression during 8 years of follow-up
Source: Eur Child Adolesc Psychiatry. 2016 Apr 22;25(11):1255–66. doi: 10.1007/s00787-016-0846-1 (PMC5083762; doi:10.1007/s00787-016-0846-1)
Supplement: Supplementary file 5 — Online Resource 5 (PDF 95 kb) [file 787_2016_846_MOESM5_ESM.pdf]

Article: Slow identification of facial happiness in early adolescence predicts onset of depression during eight years of follow-up

Journal: European Child & Adolescent Psychiatry

Authors: Charlotte Vrijen, Catharina A. Hartman, Albertine J. Oldehinkel

Affiliation: Interdisciplinary Center Psychopathology and Emotion regulation, Department of Psychiatry, University of Groningen, University Medical Center Groningen

Corresponding author: C. Vrijen@umcg.nl

Online Resource 5

Results logistic regression analyses of DSM-IV depression and symptoms of anhedonia and sadness for at least several days between age 11 and age 19 on facial emotion recognition reaction times at age 11, ***adjusted for cohort status***

		Depression[a] N=1803-1815		Anhedonia[b] with sadness as covariate N=1716-1727		Sadness[c] with anhedonia as covariate N=1718-1727	
		OR	P	OR	P	OR	P
**Emotions tested separately**	RT Happy	1.07	.29	1.04	.44	0.97	.56
	RT Sad	0.98	.69	0.93	.18	1.04	.42
	RT Angry	1.02	.81	0.99	.79	0.97	.59
	RT Fearful	0.97	.58	1.01	.82	1.04	.48
**Multi-emotion models**	RT Happy	1.18	.06	1.21	.015	Not tested	Not tested
	RT Sad	0.87	.12	0.81	.008	Not tested	Not tested
**Posthoc analyses**	RT Sad - RT Happy (HS)	0.90	.11	0.86	.007	Not tested	Not tested

[a] CIDI-based DSM-IV diagnosis of major depressive disorder, minor depressive disorder or dysthymia;
[b] Symptoms of anhedonia for at least several consecutive days;
[c] Symptoms of sadness for at least several consecutive days;
All effects were adjusted for error proportions, gender, age at the time of the facial emotion recognition task, and cohort status; OR = odds ratio; RT = mean reaction time for correct responses; all RTs in this table are standardized (Z-values) with one exception: HS was calculated on unstandardized RT Sad and RT Happy and was standardized afterwards
